# Design Reliable Bus Structure Distributed Fiber Bragg Grating Sensor Network Using Gated Recurrent Unit Network

**DOI:** 10.3390/s20247355

**Published:** 2020-12-21

**Authors:** Amare Mulatie Dehnaw, Yibeltal Chanie Manie, Ya Yu Chen, Po Han Chiu, Hung Wei Huang, Guan Wei Chen, Peng Chun Peng

**Affiliations:** Department of Electro-Optical Engineering, National Taipei University of Technology, Taipei 10608, Taiwan; t108999404@ntut.edu.tw (A.M.D.); yibeltal_chanie@ntut.edu.tw (Y.C.M.); t107658052@ntut.edu.tw (Y.Y.C.); t106650004@ntut.edu.tw (P.H.C.); t108658085@ntut.edu.tw (H.W.H.); t108658073@ntut.edu.tw (G.W.C.)

**Keywords:** Fiber Bragg Grating, Intensity Wavelength Division Multiplexing, gated recurrent unit network

## Abstract

The focus of this paper was designing and demonstrating bus structure FBG sensor networks using intensity wavelength division multiplexing (IWDM) techniques and a gated recurrent unit (GRU) algorithm to increase the capability of multiplexing and the ability to detect Bragg wavelengths with greater accuracy. Several Fiber Bragg grating (FBG) sensors are coupled with power ratios of 90:10 and 80:10, respectively in the suggested experimental setup. We used the latest IWDM multiplexing technique for the proposed scheme, as the IWDM system increases the number of sensors and allows us to alleviate the limited operational region drawback of conventional wavelength division multiplexing (WDM). However, IWDM has a crosstalk problem that causes high-sensor signal measurement errors. Thus, we proposed the GRU model to overcome this crosstalk or overlapping problem by converting the spectral detection problem into a regression problem and considered the sequence of spectral features as input. By feeding this sequential spectrum dataset into the GRU model, we trained the GRU system until we achieved optimal efficiency. Consequently, the well-trained GRU model quickly and accurately identifies the Bragg wavelength of each FBG from the overlapping spectra. The Bragg wavelength detection performance of our proposed GRU model is tested or validated using different numbers of FBG sensors, such as 3-FBG, 5-FBG, 7-FBG, and 10-FBG, separately. As a result, the experiment result proves that the well-trained GRU model accurately identifies each FBG Bragg wavelength, and even the number of FBG sensors increase, as well as the spectra of FBGs, which are partially or fully overlapped. Therefore, to boost the detection efficiency, reliability, and to increase the multiplexing capabilities of FBG sensor networks, the proposed sensor system is better than the other previously proposed methods.

## 1. Introduction

Nowadays, the Internet of Things (IoT) is becoming an extremely important communication model and has triggered numerous global research interests [1]. IoT is an influential communication paradigm that links glove-wide entire network components and interconnects with each other [1]. IoT is a state-of-the-art automation and analytics platform that uses incorporate technologies such as networking, sensing, big data, and artificial intelligence to realize an intelligent service sensing and monitoring in a wide-ranging system [1,2]. The sensor network, comprising several sensors, is used as a crucial component of the IoT framework to attain multi-point measurements for a given physical environment [1,2,3]. From a various number of sensor network categories, Fiber Bragg Grating (FBG) sensors are widely used in complex environments because of their strong anti-electromagnetic interference, slight size, corrosion resistance, high-temperature tolerance, high sensitivity, and strong multiplexing capabilities [3,4,5,6,7,8]. Additionally, they displays massive bandwidths, have few broadcast victims, allow vast topographical coverage, and can be operated without electrical powering of local batteries to decrease the risk of sparking in flammable surroundings [1,3,4]. Furthermore, the FBG sensor is an exceptional sensor element, which is appropriate to measure temperature, strain, pressure, slope, displacement, acceleration, load, and other paramters in IoT ecosystems as well as in various industrial applications for both motionless and active modes of operation [3,9,10]. To realize and measure the above-listed parameters, several FBG sensors need to be multiplexed in one cable [3,4,5,6].

In the previous scheme, wavelength division multiplexing (WDM) [11,12] and time-division multiplexing (TDM) [13,14] techniques have been proposed to increase the number of FBG sensors that are multiplexed in a single fiber cable. However, in the WDM technique, each FBG has a limited operating range, which is not able to overlap. Thus, the number of FBG sensors multiplexed in WDM is limited through the operating spectra of the respective FBG sensor and the entire bandwidth light source [11,12]. Every FBG spectral signal in the WDM of the FBG sensor network is needed to have an independent operational region, which means that the adjacent spectrum does not overlap. In addition, the TDM method [13,14] is prone to delays, and it is very difficult to guarantee service to the desired receivers. This makes the number of FBG sensors in the sensor system very restricted.

Recently, to resolve the problems of WDM and TDM, researchers have proposed intensity wavelength division multiplexing (IWDM) techniques. IWDM is used to increase the number of multiplexed FBG sensors, and it has a low-complexity advantage [3,15,16,17,18]. Several network topologies, such as tree, ring, mesh, star, and bus topologies, have been proposed in the FBG sensor network area [3,12,13,17] to implement different multiplexing techniques. However, each network topology has its drawbacks. Nevertheless, due to the simplicity of installation, cable cost reduction, and ease of fault troubleshooting, bus topology is the most widely available and preferred architecture for distributed sensor network applications [4,12,13,19] compared with the other topologies. Furthermore, because of its easily re-configurable structure, bus topology is a preferential structure over the other network topologies for incorporating many numbers of FBG sensors. IWDM-based FBG sensor network structures can be designed using one or a hybrid of one or several network topologies.

The IWDM approach allows the adjacent FBG reflection spectrum to overlap and crossover to each other without their identification being lost [3,15,16,17,18]. This realized that IWDM is used to improve the multiplexing capacity and capability of the FBG sensor system and is capable of increasing the interrogated number of sensors twice more than conventional WDM [15,16,17,18]. However, in an IWDM-based sensor system, an unmeasurable gap is created when the bandwidth of one FBG is slightly greater than the neighboring FBG, which induces crosstalk between FBGs and increasing errors in Bragg wavelength measurements [15,16,17]. Therefore, accurate determination of the FBG Bragg wavelength from the overlapping spectra is the primary task of the FBG sensor network. Recently, to boost the accuracy of Bragg wavelength measurements, several evolutionary algorithms such as genetic algorithms (GAs) [20], differential evolution (DE) algorithms [21,22], particle swarm (PSO) algorithms [23,24], and distributed estimation algorithms (EDA) [25] have been proposed. However, as the number of sensors increases, evolutionary algorithms typically need longer processing time to achieve better accuracy and have higher detection errors, which affect real-time monitoring of the Bragg wavelength measurement.

In successive works, to improve the overlap spectra detection speed and accuracy, several machine learning approaches, such as the least-squares support vector regression machine (LS-SVR) [26] and the extreme learning machine (ELM) [11,17], have been proposed. However, these proposed algorithms have a lack of adaptability and a poor learning capacity for a greater number of FBG sensor systems, and during configuration changes, these algorithms also have reusability problems. In addition, in traditional machine learning techniques, features are manually extracted and consistency is limited, while deep learning ensures that data features and spectral features are automatically extracted via the multi-layer network structure [3,14,18,27,28,29,30]. More recently, to enhance the accuracy of Bragg wavelength detection, several deep learning techniques such as long short-term memory (LSTM) [27], convolutional neural networks (CNNs) [28], back-propagation neural networks (BPNNs) [29], a hybrid of discrete-wavelet transform (DWT) and LSTM [18] techniques, and MLP [14,30] techniques have been proposed. Meanwhile, the speed of training time, accuracy of Bragg wavelength detection, and the reusability capability of the model are still problems. Therefore, the model’s reusability, accuracy, and computation time speed are still the limitations of these models as the number of FBG sensors increases.

The gated recurring unit (GRU) deep learning algorithm Bragg wavelength measurement technique fills the gap of those models’ learning efficiency, because GRU has a higher capacity to adapt data fluctuations and provide efficient generalization [3,31]. The GRU structure is relatively simple and its learning speed is fast, and it is currently one of the most effective methods studied for the spectral characteristics of overlapping FBGs [3]. It is characterized by a high capacity of learning, and understanding of complex data, and an easy adaptability to dynamic and temporal changes in data [3,31].

In this paper, we proposed GRU-based Bragg wavelength detection techniques to solve IWDM’s crosstalk problem, to improve the Bragg wavelength detection accuracy, the computational time speed, and the multiplexing capability of the FBG sensor system. In general, the contributions of this paper are described as follows:

- To design a reliable multi-point sensing bus structure FBG sensor network.
- To increase the number of FBG sensors using IWDM techniques.
- To demonstrate the promising GRU model for accurate Bragg wavelength detection of each FBG sensor from the overlapping spectra.
- To confirm the preferentiality of the proposed GRU model in Bragg wavelength detection over other traditional and recently proposed machine learning models.
- To verify the performance of the proposed FBG sensor network in terms of computational time, Bragg wavelength detection accuracy, and multiplexing capability as the number of FBG sensors in the network is increased.

The remaining portions of this paper are organized as follows: the operational principle and experimental setup is stated in Section 2; the methodology part is discussed in Section 3; and the results and discussion are presented in Section 4. Finally, in Section 5, the conclusion of this paper is presented.

## 2. Operational Principle and Experimental Setup

### 2.1. Schematic Diagram of Bus Architecture Multi-Point FBG Sensor Network

**Figure 1:**
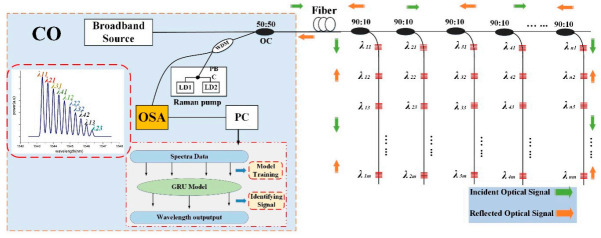
Schematic illustration of the FBG Sensor distributed node Bus architecture network.

Figure 1 illustrates the proposed schematic diagram of a IWDM-based multi-point sensing bus structure sensor network using a GRU model for enhancing the computational time speed, the accuracy of Bragg wavelength detection, and the multiplexing capability for the FBG sensor network. Moreover, within this proposed multi-point sensing bus architecture, we used IWDM techniques to increase the number of FBG sensors that multiplexed in a single fiber cable. Moreover, for signal propagation loss compensation and to recover the signal quality power degradation, we used the Raman amplification approach. As shown in Figure 1, the bus structure FBG sensor network comprises a central office (CO), a Raman amplifier, and several FBGs. The CO contains an optical spectrum analyzer (OSA), an optical coupler (OC), and an erbium-doped fiber amplifier (EDFA). The responsibility of the central office is to monitor the light source and to measure the strain Bragg wavelength s. The broadband source that generates from EDFA is transmitted through 1 × 2 OC to illuminate the FBG sensor array located in a bus structure. As shown in Figure 1, in a bus structure multi-point sensing system, FBG sensors are positioned within a branched bus structure, which is separated from the main fiber cable with the power ratio of 90∶10 OC. Thus, the main fiber cable is fragmented into five segment subnets using a 1 × 2 OC and each subnetwork supports *N* number of FBGs. The reflected FBG signals are then transmitted through the coupler to the CO and the remaining signals are transmitted to the following FBGs. Each FBG’s back-reflected light is transmitted and propagated back to the same coupling path through the other arm of a 1 × 2 OC and enters into the Raman amplifier. Through a WDM coupler, the Raman output power can be pumped to the remote single-mode fiber (SMF). Two laser diodes (LDs) with wavelengths of 1420 nm and 1450 nm constitute part of the Raman amplifier. The outputs from the two LDs are combined by the Polarization Beam Combiner (PBC) and the Raman amplifier’s amplified output is loaded into the OSA. For further data preprocessing and deep learning model training and validation, this spectral sampling data collected by OSA will be transferred to personal computer (PC).

Within the WDM technique, the FBG sensor multiplexing number is limited by the reflective range of each FBG sensor and the complete light source bandwidth. Each FBG sensor in the sensor system is allocated a specific spectral operational region and the wavelength of the adjacent FBGs is not permitted to overlap [11,12]. The multiplexing capacity of the FBG sensor system is significantly limited by these problems. On the other hand, our proposed IWDM approach requires the spectrum of neighboring FBGs to be permitted the possibility of crossing and overlapping one another within the sensor network. Therefore, within the proposed scheme, the IWDM method can simply increase the number of interrogated FBG sensors.

In the proposed bus structure IWDM-based FBG sensor system, the spectrum of the neighboring FBG sensors can be overlapped while the spectrum of one FBG is entering into the overlapping region. There are three different types of overlapping situations able to exist, such as non-overlapping spectra, partially overlapping spectra, and completely overlapping spectra of FBG sensors. If the spectra of FBGs are non-overlapping, the spectra of several FBGs are distinct and the Bragg wavelength of each FBG can be easily detected. In another way, partial and completely overlapping leads to crosstalk and increases the Bragg wavelength detection error. Specially, if each FBG spectrum is entirely overlapped with the other FBG, it is very difficult to detect or realize the exact Bragg wavelength of each FBG from the overlap spectra using conventional peak detection methods or OSA. Thus, in this paper, we proposed the GRU model to resolve the crosstalk or overlap of FBG spectral.

### 2.2. Experimental Setup of IWDM-Based Bus Structure Sensor Network

**Figure 2:**
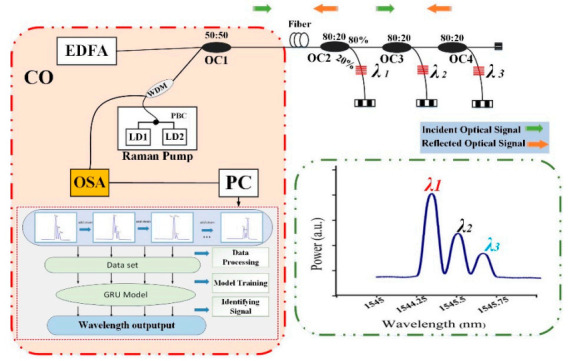
Experimental setup of IWDM based distributed node Bus architecture FBG sensor system.

The simplistic experimental setup of the proposed IWDM-based bus structure multi-point sensing FBG sensor network has been carried out as illustrated in Figure 2. To demonstrate the applicability of the proposed IWDM-based bus structure FBG sensor network scheme, we implemented the experimental step using 3 FBG sensor networks. Three identical FBGs with different wavelengths are located in a bus structure through 1 × 2 OC in this simple experiment. The FBG sensor system comprises of the component such as Raman pump, EDFA, an Optical coupler (OC), three FBG sensors, an OSA and PC. The broadband light signals emitted from EDFA propagate to FBGs over the 1 × 2 optical coupler (OC1) and split into three paths. In the beginning, one of the paths of OC1 propagates to OC2. Sequentially, OC2 is fragmented by 1 × 2 OC2 (80:20 coupler ratio) and the 20% power ratio propagates to *λ*_1_. Likewise, the remaining 80% signal propagates into the 1 × 2 OC3 (80:20 coupler ratio) and a 20% power ratio propagates to *λ*_2_. Furthermore, the 80% signal power propagates into the 1 × 2 OC4 (20:80 coupler ratio) and a 20% power ratio propagates to *λ*_3_. Likewise, the structure is opened to continue to repeat this iteration based on the demand of several FBGs. Moreover, the remaining arm of OC1 is connected to the Raman amplifier, which can regenerate the power signal to compensate for the signal loss. After amplification of the signal, the signal passes to OSA to measure and capture the spectra reflected from each FBG. Thus, to train the proposed GRU network, the training sensor data can be collected using OSA. The span width of the OSA was set to 5 nm and sampled by 2001 points, corresponding to a sample resolution of 2.5 pm. Finally, the measured signal is sent into a Personal computer (PC) for further data pre-processing and analyzing data.

Therefore, the main objective of this paper is to solve and enhance the computational time speed, detection accuracy, and multiplexing capacity for measuring the Bragg wavelength of each FBG.

## 3. Methodology

### 3.1. Dataset Collection and Data Pre-Processing

In this proposed work, we determine and understand the relationship between the strain and the reflection spectrum of FBG. During the experiment, the first task is to record the strain sensor data by applying different strain steps to the *λ*_1_ sensor. The experiment is conducted based on the setup shown in Figure 2. Thus, the training dataset and testing dataset are recorded with specified parameters in the experimental setup, including the peak power of each FBG, FWHM, the central wavelength of each FBGs, and sample points.

To increase the reliability and to prove and test the detection performance of the proposed GRU model with a different number of sensors, we collected various strain sensor data from a different number of sensors. Thus, we consider 4 cases or scenarios for collecting sensor data. Table 1 shows the four scenarios or cases such as case 1 (real experiment), case 2, case 3, and case 4 with the number of FBG sensors being 3, 5, 7, and 10, respectively. In case 1, the real data were collected based on a real experiment where the central wavelength of the *λ*_1_ sensor is shifted from 1545 nm to 1545.75 nm with 26 strain steps. While in cases 1, 3, and 4, the data were collected based on Matlab simulation using Equation (3). In all cases, the wavelength of *λ*_1_ was shifted, while the remaining FBG sensors wavelengths were fixed.

The training and testing datasets were the reflection spectra of 3, 5, 7, and 10 number of FBGs at distinct strain values of *λ*_1_ sensor. Thus, we collected a number of training datasets by applying different strain values to *λ*_1_ sensors until we found the maximum strain values. When the strain is applied to the *λ*_1_ sensor, the central wavelength of the *λ*1 sensor is shifted in the range from 1545 nm to 1545.75 nm and the measured spectra of FBGs are sampled by 2001 points, whereas the central wavelengths of other FBGs remain fixed. The strain applied to the *λ*1 sensor at each strain step is ~75 µε. A total of 26 strain steps is applied to collect the data. The central Bragg wavelength will shift if a strain is applied to *λ*_1_ sensor because of the refractive index changes. Hence, the detected wavelength of the *λ*_1_ sensor also varies. According to the longitudinal strain applied to a fiber grating, the center Bragg wavelength shift of *λ*_1_ sensor is illustrated as:

(1) $$\Delta \lambda B=\lambda B\left(1–\;Pe\right)\varepsilon$$

where Δ*λ_B_* is the difference of central Bragg wavelength shift, *λ_B_* is the central Bragg wavelength, *Pe* is the elastic-optical constant (*Pe* ≈ 0.22), and *ε* is the applied strain to *λ*. Once the central Bragg wavelength change of *λ*_1_ is defined at each applied strain value, the central Bragg wavelength of *λ*_1_ at each applied strain value can be estimated by creating a new equation from the above Equation (1):

(2) $$\lambda B\;=\;\frac{\Delta \lambda B}{\left(1–\;Pe\right)\varepsilon \;}\;\;\;$$

However, it is difficult to determine all the reflection spectrum of FBGs since the sample data recorded on OSA is vastly based on experiments. To solve such factors, we have to convert the problem into a simplified procedure to alleviate it. Accordingly, to convert the central wavelength detection problem into a regression problem, we undertake that R(λ) is the reflected spectra of all FBGs and *λ_Bi_ I* = 1, 2, 3 is the central Bragg wavelengths of the *i*th FBG. The OSA measured spectrums are expressed as follows:

(3) $$R\left(\lambda ,\lambda_{B}\right)\;=(\overset{n}{\underset{i}{\sum }}R_{i}g_{i}\left(\lambda ,\lambda_{Bi}\right))\;\;\;$$

Where the central Bragg wavelengths of FBGs are *λ_B_ = λ_B_*_1_*, λ_B_*_2_*, λ_B_*_3_*, R_i_g_i_ λ, λ_Bi_* is the peak reflectivity of the FBG *i*th, *n* is the number of FBG sensors. Hence, at each applied strain value, we can adjust the central wavelength of *λ*_1_ using Equation (3), and then the reflection spectrum of FBGs are recorded using MATLAB.

**Figure 3:**
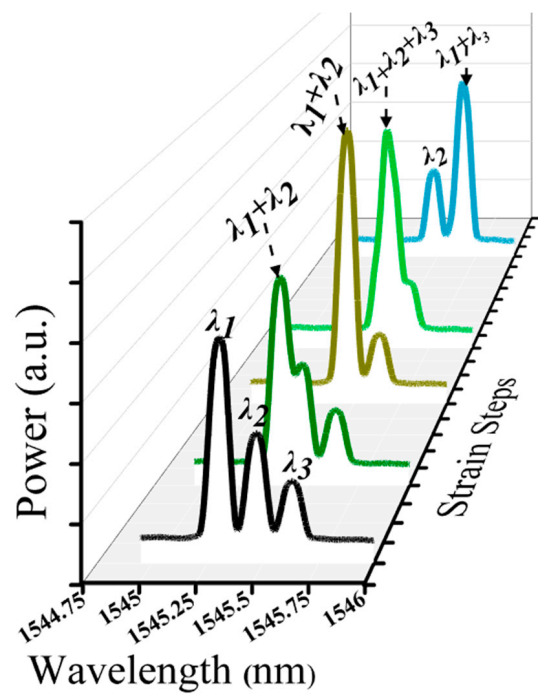
The reflected spectra of three FBGs with some applied strain to the *λ*_1_ sensor.

Figure 3 shows the sampled reflected spectra of three FBGs (case 1) at different strain steps when a strain is applied to *λ*_1_ corresponding to strain steps 0, 8, 10, 11, and 25. As illustrated in the Figure, the spectra of three FBGs at strain step 0 are distinct. Furthermore, the spectra of *λ*_1_ and *λ*_2_ sensors are partially overlapped at strain step 8. However, the spectra of *λ*_1_ and *λ*_2_ sensors, and the spectra of *λ*_1_ and *λ*_3_ sensors, are fully overlapped at strain steps 10 and 25, respectively. Furthermore, the spectra of *λ*_1_*,* *λ*_2_ and *λ*_3_ are partially overlapped at strain step 11. The overlapping FBG spectra create crosstalk and unexplained gaps between FBG sensors, which are difficult to determine the exact central (peak) wavelength using the conventional CPD technique.

The collected training data (i.e., the 3, 5, 7, and 10 FBG sensor reflection spectra at distinct strains) are denoted as Y = [*y*_1_, *y*_2_ … *y*_i_], where i is the length of the data input dimensionality. We have a total of 2001 features as input dimensionality to the proposed system. Accordingly, the entire dataset obtained in each case was divided into training and test datasets following a certain pre-processing step. A total of 20,000 training datasets and 2000 evaluation datasets have been used to train our proposed GRU-based wavelength detection system. Then, the sensor data are fed into the GRU network to measure the Bragg wavelength of each FBG. GRU can learn and understand the features from the reflection spectra of FBG sensors and can design the Bragg wavelength detection model for the FBG sensor system. Finally, the well-trained LSTM model estimates the Bragg wavelength of the unknown test samples from the test data, and a test loss is measured.

### 3.2. Gated Recurrent Unit Basic Principles

Nowadays, several researchers are continuing to use deep learning methods in various applications of regression problems because the current deep learning models are appropriate for nonlinear and complex data structures.

**Figure 4:**
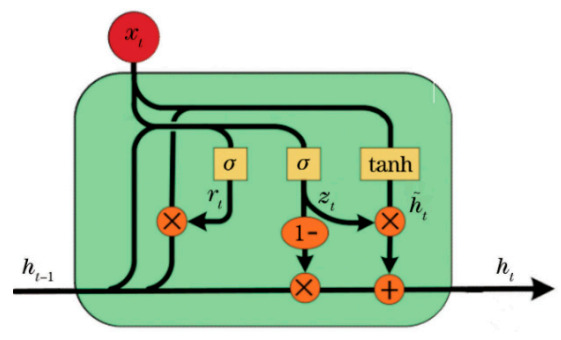
Fully connected GRU network structure.

The GRU model is a special kind of gated recurrent neural network that manages sequential data-based regression problems by deeply learning the feature of the sequential datasets [31]. GRU has been applied in various applications, such as the self-healing fiber-optic sensor approach [3], signal processing, and detection of Bragg wavelengths ([17,18,28]). GRU can also be considered as a variation on the LSTM because both are designed similarly and, in some cases, produce equally excellent results [3,31]. GRU aims to solve the vanishing gradient problem and the long-term dependency problem, which comes with a standard recurrent neural network. To solve the vanishing gradient problem of a standard RNN, GRU uses the so-called update gate and reset gate. These gates are two vectors, which decide what information should be passed to the output. The most important thing about them is that they can be trained to keep information from long ago, without washing it through time or remove information that is irrelevant to the prediction. Using its update and reset gates, the GRU network can alleviate complex problems. Figure 4 shows the internal structure of the fully connected network of the GRU unit composition. As shown in the Figure, *r_t_* is the forget get, *z_t_* is the update get, *h_t_* is the current unit, *h_t_*_−1_ hidden state of the GRU unit at time *t*, and *t* − 1, *xt* is the input at time *t*, *σ* is the Sigmoid function, and tanh is the hyperbolic tangent function, respectively. The rest gate and update gate of GRU is computed as follows:

(4) $$z_{t}=\sigma_{g}\left(W_{z}x_{t\;\;}+U_{z}h_{t-1\;\;}+b_{z}\right)$$

(5) $$r_{t}=\sigma_{g}\left(W_{r}x_{t\;\;}+U_{r}h_{t-1\;\;}+b_{r}\right)\;\;$$

(6) $$\hat{h_{t}}=\Phi_{h}\left(W_{h}x_{t\;\;}+U_{h\;}(r_{t\;\;}ʘ\;h_{t-1\;\;}\right)+b_{h})$$

(7) $$h_{t}=\left(1-z_{t\;\;}\right)\;ʘ\;h_{t-1\;\;}+z_{t}\;ʘ\;\hat{h_{t}})$$

(8) $$\sigma \left(s\right)=\frac{1}{1+\exp\left(-s\right)}\;\;$$

(9) $$tanh\left(s\right)=\frac{sinh\left(s\right)}{cosh\left(s\right)}\;\;\;=\;\;\frac{\exp\left(s\right)-exp\left(-s\right)}{\exp\left(s\right)+\exp\left(-s\right)}$$

## 4. Results and Discussion

### 4.1. Gated Recurrent Unit (GRU) Model Structure

**Figure 5:**
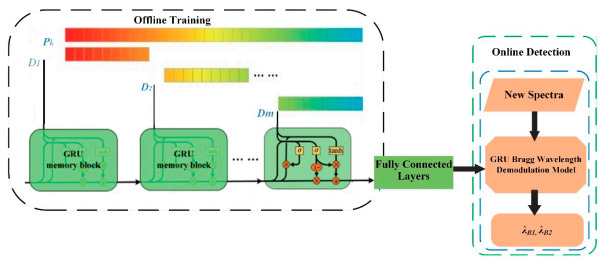
Bragg Wavelength detection architecture of the GRU model.

To implement the GRU-based Bragg wavelength detection, we used a computer with the following specifications to train the proposed model (has Intel Core *i*7-2.70 GHz CPU and 8.0 GB RAM). For accurate measurement of the Bragg wavelength of the FBG sensor network IWDM-based bus architecture, we proposed the GRU model. In this paper, the wavelength of the spectral detection problem is converted into a nonlinear regression problem, and a regression model is established using the GRU network, and the overlapped spectrum of two FBGs is realized through the model of Bragg wavelength detection. The Bragg detection process is mainly divided into two phases, namely offline learning, and online detection, as shown in Figure 5. The first is the offline learning step. In a certain order row function learning, each of the split spectrum sequences is sequentially sent to the GRU memory block. The training data set D is expressed as follows:

(10) $$D=\left(P_{1}\;,T_{1}\right),\;\ldots ,\left(P_{k},\;T_{k}\right),\ldots ,\left(P_{N},T_{N}\right)\;\;$$

Where: the spectrum of the *k^th^* FBG pair *P_k_* *∈ RI, R* is all the set of FBG pairs, *I* is the number of sampling points; *T_k_* = (*λ_B_*_1_*, λ_B_*_2_*) k,* is the Bragg wavelength of the corresponding FBGs. Consequently, the sequential features of the reflection spectra of FBGs at various strain steps are considered in the model training stage to measure the Bragg wavelength of each FBG. Once the training process is completed, the well-trained GRU model can be used in the online detection process to achieve fast and accurate Bragg wavelength detection.

### 4.2. Optimal Parameter Selection and Performance Comparison

In this proposed IWDM-based bus structure multipoint FBG sensor scheme, we used the GRU model to correctly quantify the Bragg wavelengths of each FBG. The measurement of the strain Bragg wavelength of FBGs and the entire result of the GRU model is also compared with the current other deep learning models, such as MLP and LSTM. Accordingly, several numbers of repeating pieces of training have been computed on the proposed model until we obtained the optimal results. We updated various parameters during the training of the model until the optimal solution was obtained. To perform training of our proposed model, we select the optimal values of several numbers of parameters such as epochs, hidden units, batch sizes, and optimizer and activation functions. Accordingly, optimizers are techniques used to modify the neural network features, such as weights and learning rate, to minimize losses. By minimizing the function, optimizers are used to resolve the optimization problems.

**Figure 6:**
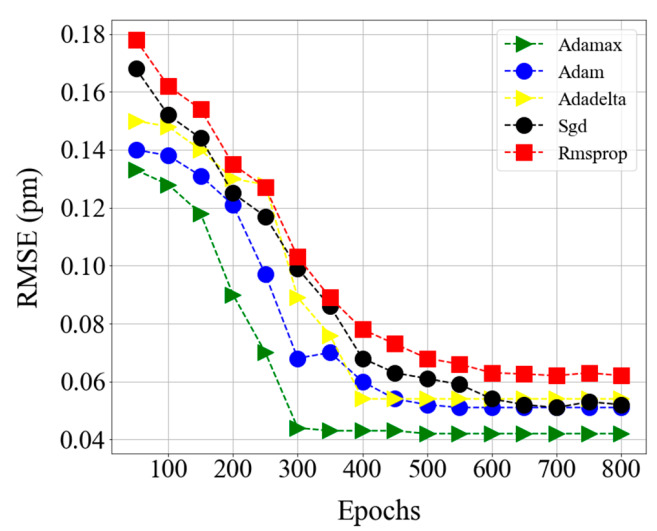
Comparison of optimizer accuracy using mean square error (MSE) at various epochs.

Optimizers are systems or methods that are responsible for reducing losses and ensuring the most precise outcomes possible. rmsprop, sgd, adadelta, Admax, and adam are some of the optimizers. Ee have seen several RMSE, MAE, MSE, and training time results by alteration of all these parameter values. As shown in Figure 6, a smaller MSE (i.e., 0.04 pm) is achieved by Adamax optimizers than by the other optimizers. Thus, the proposed model is trained using an Adamax optimizer.

Moreover, we have used several activation functions: Relu, sigmoid, tanh, and softmax to squeeze the output of the proposed model. As an outcome, the optimum values are achieved in 64 batch size, 300 epochs/iterations, 4 hidden layers, and 500 hidden units. Using the Adamax optimization algorithm and the tanh activation function, we compiled the network after many training sessions. Sequentially, the dense layer determines the outputs of the prediction, and then we used a loss function to compare the prediction outputs with the actual values. For measuring the training and validation losses of the proposed approach, MSE is the loss metric. The MSE is calculated as follows:

(11) $$MSE=\frac{1}{n}\sum_{i=1}^{n}{\left(y_{i}-y\right)}^{2}\;\;$$

Where the number of predicted values is expressed by *n*, *y_i_* denotes the actual value and *y* is the predicted value of the model results.

**Figure 7:**
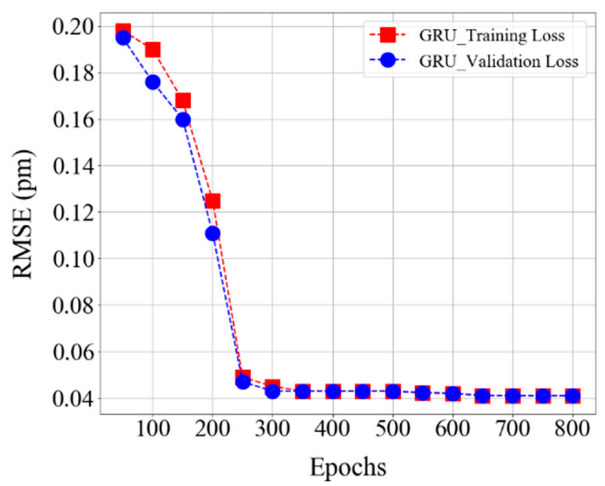
The training loss and validation loss plot of the GRU model within all cases.

Figure 7 demonstrates the loss plot and the effect of the number of iterations during the training and validation of the GRU model. The training and validation losses decrease with an increasing number of epochs. Moreover, the training and validation loss converges quickly after around 200th epochs, and the optimum value is obtained at the 300th epoch with the average error in training and validation being the same as 0.04 pm. The computational testing time and training time is 0.437 s and 413 s, respectively.

### 4.3. GRU Performance Testing and Evaluation

To validate the reusability of the proposed well-trained GRU model, we tested and verified the proposed model experiments with test data of different sensor numbers, such as 3-FBG, 5-FBG, 7-FBG, and 10-FBG. We used the same obtained optimal parameter values for testing our proposed model in different sensor numbers. The efficacy of the proposed Bragg wavelength detection model has been validated even if the adjacent FBGs are partially or completely overlapped. In separate cases, the detailed model verification was validated as follows.

#### 4.3.1. Case One: 3-FBG Sensor Network Variable Strain Effect Detection

As mentioned in Table 1, the first scenario consists of 3-FBG sensors. The working Bragg wavelength range *(λ*_1_*, λ*_2_*,* and *λ*_3_) FBGs are in between 1545 nm and 1545.75 nm. In the first scenario, the reflection spectra of the 3-FBG sensor that either partially or fully overlapped spectra were fed to our well-trained GRU model to test the performance and to check the validity of our proposed GRU model. Table 2 illustrates the actual and predicted wavelength values of *λ*_1_*, λ*_2_*,* and *λ*_3_ at different test cases, like when the two or three FBGs are non-overlapping, partially overlapping, or fully overlapping using our proposed model.

As illustrated in Table 2, the predicted result is almost the same as the actual value in all test rows. The average experimental RMS is 0.04 pm, and the highest RMS is 0.042 pm. In this case, our proposed GRU model detection speed is 0.437 s, and the longest detection time does not exceed 1 s. Hence, the proposed GRU model shows a high-precision Bragg wavelength detection efficiency even when the FBG spectra overlap.

**Figure 8:**
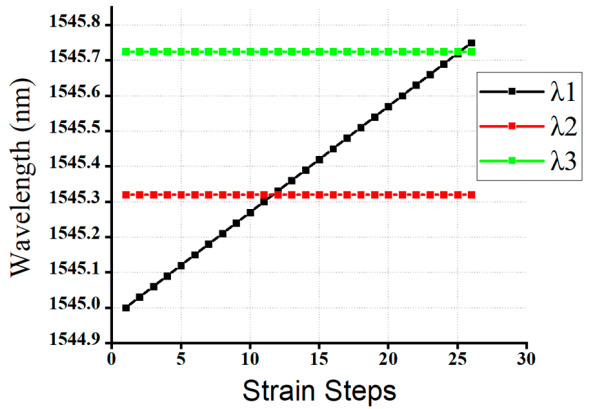
The detceted Bragg wavelength value of of 3-FBGs at different strain steps using a GRU model.

As shown in Figure 3, the reflection spectra of *λ*_1_*, λ*_2_*,* and *λ*_3_ sensors were partially or fully overlapped. Consequently, our proposed model accurately detected the Bragg wavelength of *λ*_1_*, λ*_2_*,* and *λ*_3_ sensors, even the spectra of these three FBGs are partially or fully overlapped. Moreover, Figure 8 illustrated that the detection results were obtained by the GRU model when the number of FBG sensors is three. As shown in the figure, the proposed model has the capability to detect the Bragg wavelength of each FBG even when the spectra of FBGs is partially overlapped (see strain steps 8, 9, 11, 24, 26) and fully overlapped (see strain step 10 and 25). The output of the Bragg wavelength of *λ*_2_ and *λ*_3_ is a horizontal straight line since the strain applied to *λ*_2_*,* and *λ*_3_ sensors are fixed during *λ*_1_’s stain effect, while the spectra of the *λ*_2_ sensor shifts linearly due to the applied strain. Therefore, our proposed GRU model can accurately measure the Bragg wavelength of each FBG with high accuracy in the case of fully or partially overlapping spectra.

#### 4.3.2. Case Two: 5-FBG Sensor Network Variable Strain Effect Detection

The efficacy of the GRU model has been demonstrated by increasing the number of FBG sensors connected to the proposed scheme. In the second scenario, the reflection spectra of the 5-FBG sensor that either partially or fully overlapped spectra were feeding to our well-trained GRU model to test the performance and to check the validity of our proposed GRU model. As mentioned in Table 1, the range of the central wavelength of the *λ*_1_ sensor is between 1545.3 nm and 1546.3 nm with 45 strain steps.

The Bragg wavelength detection results of our proposed GRU model have been summarized above in Table 3. As shown in the table, *λ_B_*_1_*, λ_B_*_2_*, λ_B_*_2_*, λ_B_*_3_*, λ_B_*_4_*,* and *λ_B_*_5_ are the actual values, and *λ*_1_*, λ*_2_*, λ*_3_*, λ*_4_*,* and *λ*_5_ are the detection results of our proposed GRU model. As can be seen from Table 3, the actual Bragg wavelength value of *λ*_1_*, λ*_2_*, λ*_3_*, λ*_4_*,* and *λ*_5_ sensors are almost the same as the detected Bragg wavelength of *λ*_1_*, λ*_2_*, λ*_3_*, λ*_4_*,* and *λ*_5_ sensors using our proposed GRU model.

The Bragg wavelength detection results of our proposed GRU model have been summarized above in Table 3. As shown in the table, *λ_B_*_1_*, λ_B_*_2_*, λ_B_*_2_*, λ_B_*_3_*, λ_B_*_4_*,* and *λ_B_*_5_ are the actual values, and *λ*_1_*, λ*_2_*, λ*_3_*, λ*_4_*,* and *λ*_5_ are the detection results of our proposed GRU model. As can be seen from Table 3, the actual Bragg wavelength value of *λ*_1_*, λ*_2_*, λ*_3_*, λ*_4_*,* and *λ*_5_ sensors are almost the same as the detected Bragg wavelength of *λ*_1_*, λ*_2_*, λ*_3_*, λ*_4_*,* and *λ*_5_ sensors using our proposed GRU model.

**Figure 9:**
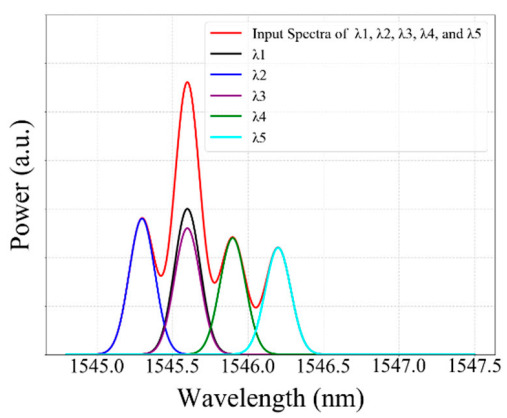
The measured Bragg wavelength of 5-FBGs sensors using a GRU model (Scenario 2).

As illustrated underneath in Figure 9, sensors *λ*_1_*,* and *λ*_3_ were fully overlapped, while the spectra of *λ*_2_*, λ*_4_*,* and *λ*_5_ are non-overlapping with other FBGs. Consequently, our proposed model accurately detected the Bragg wavelength of *λ*_1_*, λ*_2_*, λ*_3_*, λ*_4_*,* and *λ*_5_ sensors, even the spectra of these five FBGs are partially or fully overlapped.

**Figure 10:**
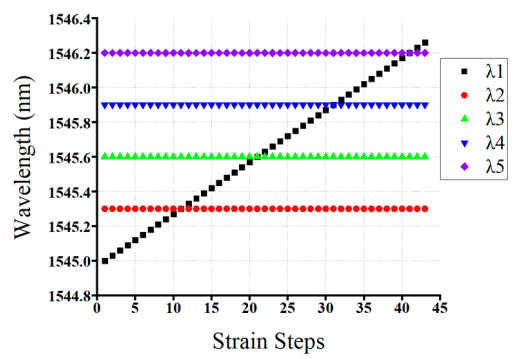
The detceted Bragg wavelength value of 5-FBGs at different strain steps using the GRU model.

Moreover, Figure 10 above illustrated that the detection results obtained by the GRU model when the number of FBG sensors is five. As shown in the figure, the proposed model accurately detects the Bragg wavelength of each FBG even when the spectra of FBGs are partially overlapped (see strain steps 10, 12, 20, 22,30,32, 40, and 42) and fully overlapped (see strain step 11, 21, 31, and 41). Therefore, our proposed GRU model can accurately measure the Bragg wavelength of each FBG with high accuracy in the case of fully or partially overlapping spectra.

#### 4.3.3. Case Three: 7-FBG Sensor Network Variable Strain Effect Detection

In the third scenario, the reflection spectra of the 7-FBG sensor that either partially or fully overlapped spectra were feeding to our well-trained GRU model to test the performance and to check the validity of our proposed GRU model. Our proposed GRU model predicts the bragg wavelength of each FBG from the partially or completely overlapped spectra. Thus, when the reflection spectrum of *λ*_1_ is partially or completely overlapped with *λ*_2_*, λ*_3_*, λ*_4_*, λ*_5_*, λ*_6_*,* and *λ*_7_ sensors, the proposed model accurately predicts the Bragg wavelength of *λ*_1_*, λ*_2_*, λ*_3_*, λ*_4_*, λ*_5_*, λ*_6_*,* and *λ*_7_ sensors. Thus, the detected Bragg wavelength of *λ*_1_, *λ*_2_, *λ*_3_, *λ*_4_, *λ*_5_, *λ*_6_, and *λ*_7_ sensors are 1546.37 nm, 1545.21 nm, 1545.4 nm, 1545.6 nm, 1545.8 nm, 1546 nm, and 1546.2 nm, respectively, and the actual Bragg wavelength of *λ*_1_*, λ*_2_*, λ*_3_*, λ*_4_*, λ*_5_*, λ*_6_*,* and *λ*_7_ sensors are 1546.32 nm, 1545.18 nm, 1545.34 nm, 1545.56 nm, 1545.73 nm, 1546.05 nm, and 1546.12 nm, respectively. Therefore, our proposed GRU model can accurately measure the Bragg wavelength of each FBG with high accuracy in the case of fully or partially overlapping spectra.

**Figure 11:**
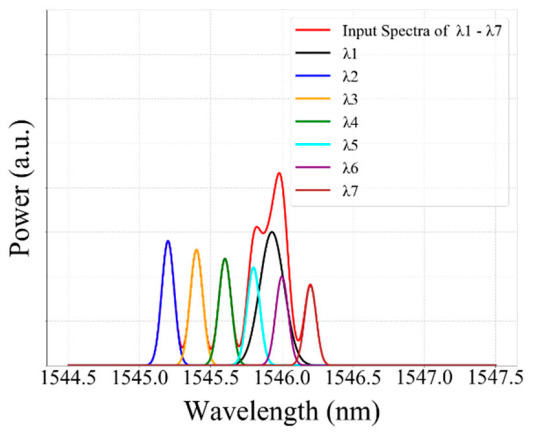
The measured Bragg wavelength of 7-FBGs sensors using a GRU model (Scenario 3).

As showed below in Figure 11, the reflection spectra of *λ*_1_*, λ*_5_*, λ*_6_ sensors were partially overlapped, while the spectra of *λ*_2_*, λ*_3_*, λ*_4_*,* and *λ*_7_ sensors are non-overlapping with other FBGs. Consequently, our proposed model accurately detected the Bragg wavelength of *λ*_1_*, λ*_2_*, λ*_3_*, λ*_4_*, λ*_5_*, λ*_6_*,* and *λ*_7_ sensors, even the spectra of these FBGs are partially or fully overlapped.

**Figure 12:**
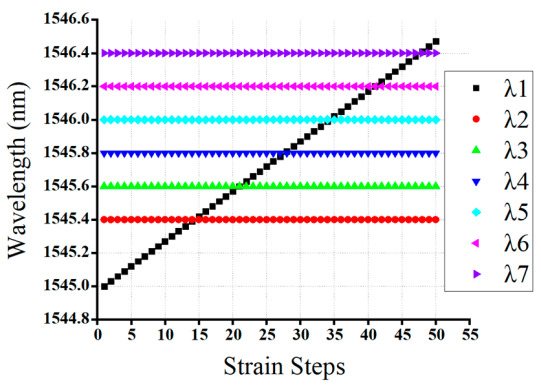
The detceted Bragg wavelength value of 7-FBGs at different strain steps using the GRU model.

Moreover, Figure 12 illustrates the detection results obtained by the GRU model when the number of FBG sensors is seven. As shown in the Figure, the proposed model has the capability to detect the Bragg wavelength of each FBG even when the spectra of FBGs are partially overlapped (see strain steps 14, 16, 21, 23, 27, 29, 34, 36, 41, 43,46 and 48) and fully overlapped (see strain step 15, 22, 28, 35, 42 and 47).

#### 4.3.4. Case Four: 10-FBG Sensor Network Variable Strain Effect Detection

In the fourth scenario, the reflection spectra of the 10-FBG sensor that either partially or fully overlapped spectra are fed to our well-trained GRU model to test the performance and to check the validity of our proposed GRU model. Our proposed GRU model predicts the Bragg wavelength of each FBG from the partially or completely overlapped spectra. Thus, when the reflection spectrum of *λ*_1_ is partially or completely overlapped with *λ*_2_*, λ*_3_*, λ*_4_*, λ*_5_*, λ*_6_*, λ*_7,_ *λ*_8_*, λ*_9_*,* and *λ*_10_ sensors, the proposed model accurately predicts the Bragg wavelength of each sensor. Thus, the detected Bragg wavelength of *λ*_2_*, λ*_3_*, λ*_4_*, λ*_5_*, λ*_6_*, λ*_7_, *λ*_8_*, λ*_9_*,* and *λ*_10_ sensors are 1546.63 nm, 1545.21 nm, 1545.4 nm, 1545.6 nm, 1545.8 nm, 1546 nm, 1546.2 nm, 1546.4 nm, 1546.6 nm, and 1546.8 nm, respectively, and the actual Bragg wavelength of *λ*_2_*, λ*_3_*, λ*_4_*, λ*_5_*, λ*_6_*, λ*_7_, *λ*_8_*, λ*_9_*,* and *λ*_10_ sensors are 1546.56 nm, 1545.13 nm, 1545.34 nm, 1545.65 nm, 1545.84 nm, 1546.14 nm, 1546.26nm, 1546.47 nm, 1546.65 nm, and 1546.86 nm, respectively. Therefore, our proposed GRU model can accurately measure the Bragg wavelength of each FBG with a high accuracy in the case of fully or partially overlapping spectra.

**Figure 13:**
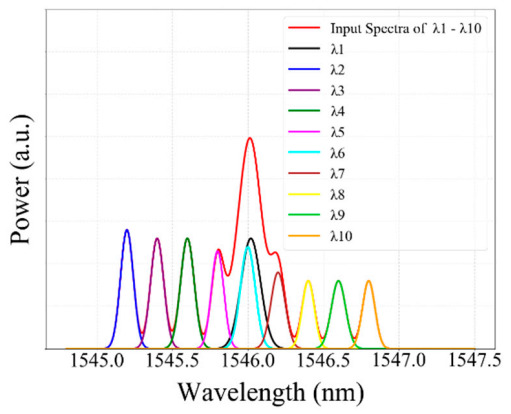
The measured Bragg wavelength of 10-FBGs sensors using a GRU model (Scenario 4).

As shown in Figure 13, the reflection spectra of *λ*_1_*, λ*_6_*,* and *λ*_7_ sensors are partially overlapped, while the spectra of *λ*_2_*, λ*_3_*, λ*_4_*, λ*_5_*, λ*_8_*, λ*_9_*,* and *λ*_10_ sensors are non-overlapping with other FBGs. Consequently, our proposed model accurately detected the Bragg wavelength of *λ*_1_ *λ*_2_*, λ*_3_*, λ*_4_*, λ*_5_*, λ*_6_*, λ*_7_, *λ*_8_*, λ*_9_*,* and *λ*_10_ sensors, and even the spectra of these FBGs are partially or fully overlapped.

**Figure 14:**
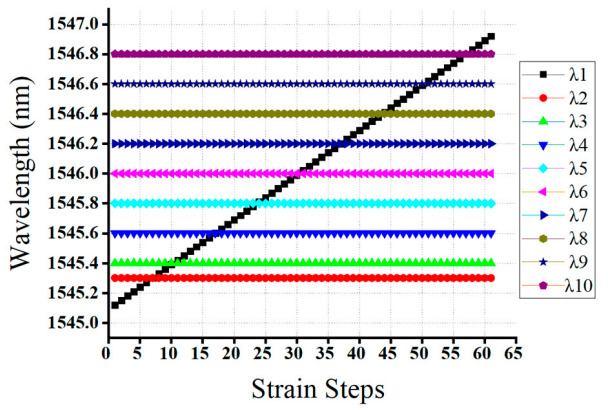
The detected Bragg wavelength value of 10-FBGs at different strain steps using a GRU model.

Figure 14 shows the detection results obtained by the GRU model when the number of FBG sensors is seven. As shown in the figure, the proposed model capable of detecting the Bragg wavelength of each FBG, even when the spectra of FBGs are partially overlapped (see strain steps 7, 11, 13, 16, 18, 21, 24, 29, 31, 36, 38, 43, 45, 49, 51, 56 and 58) and fully overlapped (see strain step 8, 12, 17, 23, 30, 37, 44, 50 and 57). Moreover, the average RMS errors of our proposed GRU model for the four different cases, such as 3-FBG, 5-FBG, 7-FBG, and 10-FBG, are summarized in Table 4. Thus, the average RMS errors of case 1, case 2, case 3, and case 4 are 0.0411 pm, 0.0421 pm, 0.0431 pm, and 0.0433 pm, respectively.

As shown in the table, the average RMS errors are less than 1 pm in all cases, even when the number of FBG sensors is 10. Besides, our proposed GRU model detectes the Bragg wavelength within a fraction of a second, which is rarely affected by the FBG number. The average RMS error and testing time is 0.0433 pm and 0.445 s, even when the number of the FBG sensor is 10-FBG sensors.

**Figure 15:**
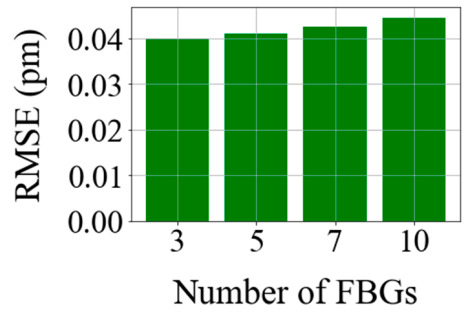
RMS error and testing time of the wavelength detection for different numbers of FBGs.

From the table, we can prove that as the sensor number increases, the RMS error also increases. Furthermore, Figure 15 shows the RMSE versus the number of sensors. As shown in the figure, when the number of FBG increases, the RMS error also increases.

**Figure 16:**
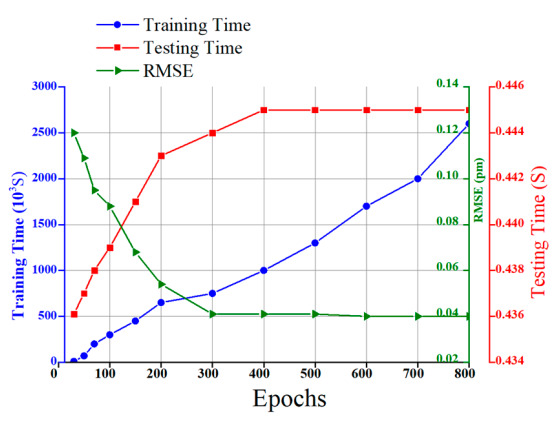
RMS error, training time, and testing time of GRU models with a different number of epochs.

Furthermore, the performance of the proposed GRU model is evaluated in terms of RMSE, training time, and testing time within the different number of epochs, as shown in Figure 16.

As shown in the figure, the RMS errors dramatically decreased, and the training and test time increased when the number of epochs increased. The RMSE and test time can be becoming stable after epoch numbers of 400. Therefore, the proposed GRU model achieves a smaller RMSE and a shorter training and test time than the previously reported work.

### 4.4. Comparison of the Model

Several Bragg wavelength detection methods have been proposed in the introduction part, such as the differential evolution DE method [21,22], a tree-search dynamic multi-swarm particle swarm algorithm (TS-DMS-PSO) [23], particle swarm optimization-based simulated annealing (PSO-SA) [24], a search tree-based Least Square Support Regression [26], Genetic Algorithm, GA [20], distributed estimation algorithm (EDA) [25], and ELM [11]. Thus, in this section, we compare and contrast the performance of our proposed GRU model with previously proposed algorithms and machine learning techniques, as shown in Table 5. As illustrated in Table 5, the Bragg wavelength detection RMS error of the proposed GRU model is substantially lower than ELM, DE, EDA, LS-SVR, TS-DMS-PSO, PSO-SA, and GA.

The overall RMS error of our proposed GRU model is 0.043 pm. Compared to the state-of-the-art techniques, the proposed GRU model offers better Bragg wavelength detection prediction efficiency. In addition, its testing speed is significantly better than all the GA, PSO-SA, TS-DMS-PSO, LS-SVR, ELM, EDA, and DE algorithms. However, the previously reported shallow learning methods showed poorer performance than the proposed GRU model due to a lack of adaptability and a low learning capacity in scalable fiber sensor environments. Furthermore, shallow learning algorithms can easily be influenced by environmental and temperature influences. Moreover, the performance of our proposed GRU model was compared with the most recent deep learning and machine learning techniques such as LSTM [18,27] and MLP [14,30], respectively. These three models were compared using the same dataset sample size, environment, and optimization parameters.

**Figure 17:**
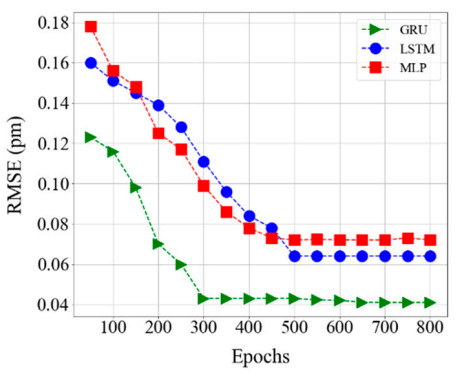
Comparison of the various model using Root Mean Square Error (RMSE) and the number of epochs.

As shown in Figure 17, the RMSE value of the GRU model is less than MLP and LSTM models within a different number of epochs. The RMSE value of GRU, LSTM, and ELM models at different test cases are summarized in Table 6. Furthermore, as the key parameter next to the RMSE, we compared the testing time performance of GRU, LSTM, and ELM models. As seen in Table 6, the maximum detection time of GRU, LSTM, and MLP, are 0.45 s, 0.98 s, and 2.5 s, respectively. Therefore, the proposed GRU model has a better computation time than other deep learning models.

In general, our proposed GRU model has a greater consistency than the evolutionary algorithm and other machine learning algorithms listed in Table 5. Therefore, in terms of reusability and flexibility, our proposed GRU model is preferable over other shallow learning algorithms.

## 5. Conclusions

This paper is focused to design and demonstrate IWDM-based Bus structure FBG sensor network schemes using the GRU deep learning model to solve the overlap problem of the FBG sensor system. We used IWDM techniques to increase the multiplexing capacity of the FBG sensor system. A GRU model is proposed for Bragg wavelength detection of each FBG from the overlap spectra. We used the reflection spectra of FBGs at different strain steps to train the proposed GRU model. Then, we train the proposed GRU network several times by adjusting different parameters until the optimal result was achieved. The detection performance of our proposed GRU model has been tested using different scenarios, such as when the number of FBG sensors are 3, 5, 7, and 10 sensors.

The experimental results showed that even if the spectra of three or more FBGs partially or entirely overlap, our proposed GRU model can accurately predict the Bragg wavelength of each FBG. When compared with the previously reported shallow learning algorithms, the proposed GRU model achieved a higher Bragg wavelength detection accuracy under the different scenarios. Moreover, the proposed GRU model has a faster computational time than ELM, LSTM, LS-SVR, and other machine learning algorithms. Therefore, our proposed FBG sensor system has a great potential to increase the number of FBG sensors in the sensor system, improves the Bragg wavelength detection accuracy, and increases the signal transmission distance of the FBG sensor network.

## Figures and Tables

**Table 1:** List of cases for test the detection performance of the proposed model with a different number of FBG sensors.

Scenarios or Cases	Number of FBG Sensors	Number of Strain Steps	*λ*_1_ Wavelength Shift Value
1 (Experimental setup)	3	26	1545 nm–1545.75 nm
2	5	45	1545 nm–1546.3 nm
3	7	51	1545 nm–1546.5 nm
4	10	63	1545 nm–1546.9 nm

**Table 2:** Results GRU model 3-FBG sensor network for randomly overlapped cases with Root Mean Square Error (MSE). “St. S.” = “Strain steps”.

St. S.	Actual Wavelength Values			Predicted Wavelength Values			RMSE/pm	Test Time/s
	$\lambda_{B1}/nm$	$\lambda_{B2}/nm$	$\lambda_{B3}/nm$	$\lambda_{1}/nm$	$\lambda_{2}/nm$	$\lambda_{3}/nm$		
1	1545	1545.32	1545.642	1545.03	1545.322	1545.643	0.042	0.439
6	1545.277	1545.32	1545.642	1545.35	1545.33	1545.644	0.04	0.432
11	1545.5	1545.32	1545.642	1545.533	1545.34	1545.643	0.04	0.437
15	1545.685	1545.32	1545.642	1545.693	1545.34	1545.643	0.04	0.437
23	1545.725	1545.32	1545.642	1545.743	1545.31	1545.643	0.041	0.438

**Table 3:** Results of the GRU model 5-FBG sensor network for randomly overlapped cases with Root Mean Square Error (MSE). “St. S.” = “Strain steps”.

St. S.	Actual Wavelength Values					Predicted Wavelength Values					RMSE/ pm
	$\lambda_{B1}/nm$	$\lambda_{B2}/nm$	$\lambda_{B3}/nm$	$\lambda_{B4}/nm$	$\lambda_{B5}/nm$	$\lambda_{1}/nm$	$\lambda_{2}/nm$	$\lambda_{3}/nm$	$\lambda_{4}/nm$	$\lambda_{5}/nm$	
22	1545.81	1545.3	1545.6	1545.9	1546.2	1545.86	1545.32	1545.62	1545.94	1546.21	0.05
28	1545.99	1545.3	1545.6	1545.9	1546.2	1546	1545.4	1545.65	1545.91	1546.21	0.04
32	1546.11	1545.3	1545.6	1545.9	1546.2	1546.15	1545.5	1545.61	1545.92	1546.21	0.04
35	1546.2	1545.3	1545.6	1545.9	1546.2	1546.23	1545.33	1545.61	1545.91	1546.22	0.04
38	1546.29	1545.3	1545.6	1545.9	1546.2	1546.32	1545.4	1545.65	1545.91	1546.22	0.04

**Table 4:** Mean RMS Error and Testing Time, Bragg Wavelength, and Detection in different cases.

Scenarios or Cases	Average RMSE (pm)	Average Testing Time (s)
1 (Experimental setup)	0.0411	0.437
2	0.0421	0.439
3	0.0432	0.441
4	0.0433	0.445

**Table 5:** Comparison of various models using Root Mean Square Error (RMSE) and testing time.

Method	RMSE (pm)	Testing Time (S)	Data Source
GRU	0.043	0.44	This work
DE	<0.2	Not Available	Reference [22]
ELM	0.918	215	Reference [11]
DE	<1	450	Reference [21]
EDA	3.0	Not Available	Reference [25]
LS-SVR	3.955	0.578	Reference [26]
TS-DMS-PSO	8.01	84.85	Reference [23]
PSO-SA	7.32	Not Available	Reference [24]
GA	301.82	1026.8	Reference [20]

**Table 6:** Comparison of the various model using Root Mean Square Error (RMSE) and Testing Time, Bragg Wavelength, and Detection in different cases.

Scenarios or Cases	Comparison of the Three Models					
	GRU		LSTM		MLP	
	Average RMSE (pm)	Average Testing Time (s)	Average RMSE (pm)	Average Testing Time (s)	Average RMSE (pm)	Average Testing Time (s)
1 (Experimental setup)	0.0411	0.437	0.075	0.52	0.081	0.9
2	0.0421	0.439	0.076	0.63	0.088	0.92
3	0.0432	0.441	0.088	0.75	0.099	1.9
4	0.0433	0.445	0.099	0.98	0.102	2.5
